# Children’s Views and Experiences of Treatment Adherence and Parent/Child Co-Management in Eczema: A Qualitative Study

**DOI:** 10.3390/children8020158

**Published:** 2021-02-20

**Authors:** Emma Teasdale, Katy Sivyer, Ingrid Muller, Daniela Ghio, Amanda Roberts, Sandra Lawton, Miriam Santer

**Affiliations:** 1 Primary Care, Population Sciences and Medical Education, Faculty of Medicine, University of Southampton, Southampton SO17 1BJ, UK; i.muller@soton.ac.uk (I.M.); or d.ghio@salford.ac.uk (D.G.); m.santer@soton.ac.uk (M.S.); 2 Centre for Clinical and Community Applications of Health Psychology, University of Southampton, Southampton SO17 1BJ, UK; k.a.j.sivyer@soton.ac.uk or; 3 Department of Psychology, University of Portsmouth, Portsmouth PO1 2UP, UK; 4 School of Health and Society, Allerton Building, University of Salford, Manchester M6 6PU, UK; 5 Centre of Evidence Based Dermatology, University of Nottingham, Nottingham NG7 2RD, UK; amandaroberts@ntlworld.com; 6 Department of Dermatology, Rotherham NHS Foundation Trust, Rotherham, S60 2UD, UK; sandra.lawton1@nhs.net

**Keywords:** children, co-management, eczema, qualitative, treatment adherence

## Abstract

Eczema affects one in five children and can have a substantial impact on quality of life. This qualitative study aimed to explore children’s views and experiences of eczema and what may affect treatment adherence from their perspective. We conducted semi-structured, face-to-face interviews with children with eczema aged 6–12 years from March to July 2018. Interviews were transcribed verbatim and analysed using inductive thematic analysis. We found that children do not typically view eczema as a long-term condition, and topical treatments (predominately emollients) were seen to provide effective symptom relief. Uncertainty around co-managing at home was expressed as children typically felt that parental reminders and assistance with applying different types of topical treatments were still needed. For some children, eczema can be difficult to manage at school due to a lack of convenient access and appropriate spaces to apply creams and psychosocial consequences such as attracting unwanted attention from peers and feeling self-conscious. Treatment adherence could be supported by reinforcing that eczema is a long-term episodic condition, providing clear information about regular emollient use, practical advice such as setting reminders to support co-management at home, and working with schools to facilitate topical treatment use when necessary.

## 1. Introduction

Atopic eczema is a long-term episodic skin condition [1], typically starting before the age of 4 years and recurring periodically throughout childhood [2]. It affects around one in five children [3] and can have a substantial impact on quality of life and cause distress to children and families because of itch, sleep disturbance and bleeding skin [4,5]. Although eczema can improve with age, for many it persists into adolescence or adulthood [6,7].

Eczema self-management introduces complex behaviours into family daily life including long-term use of topical treatments, avoidance of triggers (e.g., soap) and strategies to reduce scratching. Most children benefit from two types of topical treatments: emollients and topical corticosteroids. Emollients are non-cosmetic, medical moisturisers, which need to be applied at least daily to decrease moisture loss and protect the skin against irritants. Regular emollient use is necessary to maintain symptom control and reduce eczema flare ups [8]. Some children require multiple applications of emollients each day or before or after activities such as swimming. Different emollient products are available ranging from ointments, gels, creams and lotions, and choice will be based on eczema severity and child/parent preference. Topical corticosteroids (TCS) are creams or ointments used on a short-term intermittent basis to reduce inflammation and control flare ups [8].

One of the most common causes of treatment failure in eczema is under use of topical treatments [8]. Non-adherence to topical treatments is common in many long-term skin conditions, particularly for TCS [9]. The common-sense model of health and illness [10,11] suggests that people construct cognitive and emotional representations around the identity, cause, duration, consequences and controllability of the condition, and that treatment adherence is related to people’s understanding of their condition and its treatments, as well as perceived need for treatments and concerns about any negative effects [12]. There has been little research exploring children’s representations of long-term conditions such as eczema and their treatment [13,14].

Much of the research in childhood eczema has focused on parental understanding, beliefs and concerns about eczema and its treatment and how this affects treatment adherence. Reviews of quantitative and qualitative literature suggests eczema treatment adherence from the parents’ perspective may be challenging due to misperceptions about the long-term nature of eczema, concerns about the perceived risk of TCS and constituents of emollients, uncertainty about when and how to use topical treatments, conflicting or insufficient advice from different health professionals regarding how to use TCS, child resistance and treatment burden [15,16,17,18].

Systematic efforts to examine and explore young people’s perspectives in other long-term conditions (asthma, diabetes, arthritis) suggest that treatment adherence barriers and facilitators may differ from parents, with physical well-being, striving for normality, consideration of stigma or opinions of peers, conflict with parents and treatment complexity highlighted as important factors for young people [19,20,21].

Previous qualitative research amongst children with eczema has predominantly focused on the psychosocial impact of eczema [22,23,24]. One qualitative study exploring children’s experiences of using silk garments for the treatment of eczema [25], highlighted children’s frustration with needing to use topical treatments and a sense of disappointment that their hopes and expectations for a “miracle cure” had not been met by using the silk garments. Two qualitative studies exploring young people’s experiences of eczema treatments and how they adapt to living with eczema [26,27] found that young people (aged 12 years and over) find it challenging looking after their skin due to the episodic nature of eczema and would like treatments to have a faster and more persistent effect, suggesting that adopting the concept of “control” rather than “cure” of eczema may be challenging for young people. However, little is known about younger children’s experiences of eczema and topical treatment and what may help or hinder treatment adherence from their perspective.

Whilst younger school-aged children are likely to depend on their parents for managing eczema, it will also be a time of transition towards co-management and increasing autonomy (taking responsibility for treatment adherence). Factors that influence the gradual shift in responsibility from parents to children remain relatively unexplored within the treatment adherence literature [28]. Current research suggests parents’ perceptions of their child’s readiness, wellness and competence as well as parents’ anxiety/fear of poor health outcomes from relinquishing control may impact on this process of transition [29,30]. Again, little is known about this from the child’s perspective and little is known in the context of eczema.

Greater insight into children’s understandings of eczema and its treatment, and reasons for treatment non-adherence from the child’s perspective may help to improve outcomes for children with eczema. In addition, seeking to understand children’s views and experiences of co-managing their eczema may highlight ways to support their transition to greater independence. We aimed to explore children’s views and experiences of eczema and eczema treatments and gain insight into barriers and facilitators to treatment adherence and effective parent/child co-management from children’s perspectives.

## 2. Materials and Methods

### 2.1. Study Design

We conducted a qualitative study comprising semi-structured, face-to-face interviews with children aged 6–12 years to explore eczema and eczema management from their perspective.

### 2.2. Participants and Recruitment Procedures

Children aged 6 to 12 years with eczema were invited to take part in a qualitative interview through primary care and secondary care in England. This qualitative study formed part of a larger programme of research called Eczema Care Online (ECO) that is developing and testing online resources for parents/carers of children with eczema [31]. As part of the development phase of the ECO programme, parents/carers of children with a recorded diagnosis of eczema were invited to take part in either a qualitative interview study and/or questionnaire survey about their experiences of managing eczema. Additionally, to recruit for this study, parents/carers of children aged 6–12 years were invited to share an age-appropriate participant information sheet with their child offering them the opportunity to take part in an interview about their own experiences of eczema.

We posted a study invitation pack (invitation letter, parent and child participant information sheets, questionnaire and reply slip) from 16 general practice (GP) surgeries and clinical staff gave out a study invitation pack in Dermatology clinics in three NHS hospital trusts across South West England. GP surgeries and hospitals were identified via the Wessex Clinical Research Network (CRN) and recruited from areas of differing levels of social deprivation (as defined by Public Health England [32]) in order to identify participants with a range of views. Parents/carers were asked to return the reply slip to the study team to express their child’s interest in participating in an interview. They also reported their child’s age, gender, geographical location and eczema severity (mild/moderate/severe) on the reply slip. We obtained ethical approval for this qualitative study from the Wales REC 7 Ethics committee (REC 17/WA/0329). From now on, we will refer to parents/carers collectively as parents.

### 2.3. Organising Qualitative Interviews

We purposively sampled participants based on age, gender, eczema severity and geographical location to achieve a maximum variation sample. We sought written consent from the parent and written assent from the child prior to carrying out interviews, all of which were conducted in participants’ homes. We explained that they did not have to take part and could stop at any time. Interviews typically lasted around 30 min (range 19–46 min). Parents were invited to be present during the interviews to support their child, but we explained that we wanted to find out about the child’s perspective, and we ensured that all questions were directed to the child. All children who took part in an interview received a GBP 10 voucher. They were also offered a colouring pencil set and a toy of the study mascot (the ECO whale) at the end of the interview.

### 2.4. Conducting Qualitative Interviews with Child Participants

Two authors (ET and DG) conducted face-to-face interviews. We used a semi-structured interview guide developed by the research team and piloted amongst patient representatives to guide the interviews (Box 1). It comprised open-ended questions phrased appropriately for school-aged children to explore how children’s beliefs about eczema and treatment relate to self-management [33] and treatment adherence [12]. We used a range of developmentally appropriate and individualised techniques to help the child feel at ease and feel able to share their experiences including adopting a conversational style and focusing on what the child was doing [34,35].

Box 1 **Interview Topic Guide.**

**About eczema**

How would you describe your eczema to your friends?Can you tell me what is it like having eczema?What do you think makes eczema worse?

**About eczema treatments**

What kind of things do you do to help make your eczema better?Can you tell me what you find easy/difficult about that?Who helps you with that? At home, at school? What is easy/difficult about this?*If self-managing,* how are you finding looking after your eczema? Or how would you feel about looking after your eczema? Would you like to do more?Do you do anything else to help make your eczema better?Can you tell me what you find easy/difficult about that?

**About eczema information**

What kinds of information have you had on eczema? From where?Do you use a computer?How would you feel about using a website for information and support on eczema?What sort of things would you like to see on the website?What do you think would be helpful or would have been helpful to you in the past?What do you think would be good/not good about videos on eczema?

At the start of the interview, we initiated a conversation about the child’s interests, which often prompted them to show the researchers their favourite toys, books, stories they had written and certificates/awards they had won. We adopted a Mosaic approach [36] to data collection that involved using multiple creative and fun participatory activities [37] such as using picture cards related to the interview questions to guide the conversation (Box 2) and general play activities. Initially we introduced the children to the ECO whale toy (study mascot), colouring pencil set and ECO whale drawing and colouring sheets. We also offered stickers and Lego bricks to play with (Box 3). We aimed to adopt the “least adult role” to encourage active participation in the interview [38]. We did this by sitting on the floor with the child and engaging in their chosen activities (e.g., helping build Lego, choosing stickers or colouring pencils), and allowing the children to “direct the research agenda” by choosing to use pictures cards, starting the digital recorder or to express their views by drawing their responses to some questions (Box 4). We were flexible in our approach and found that with younger participants (6–9 years), we often talked about their eczema whilst they continued with their chosen activity. Children aged 10–12 years were happy to be asked questions in a more traditional interview format and did not feel the need to stay engaged in another activity.

Box 2 **Example picture cards used alongside topic guide.**

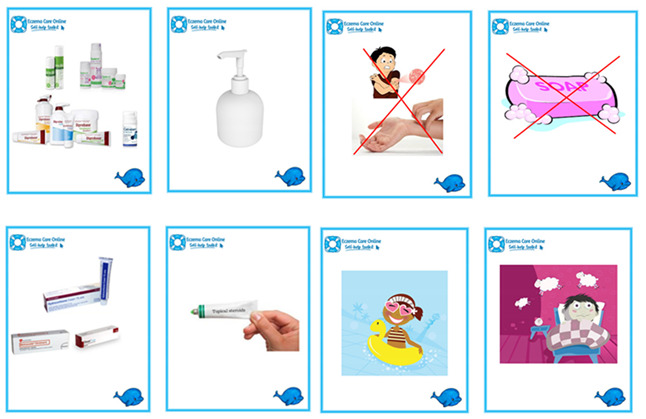

Box 3 **Examples of play activities adopted in child interviews.**

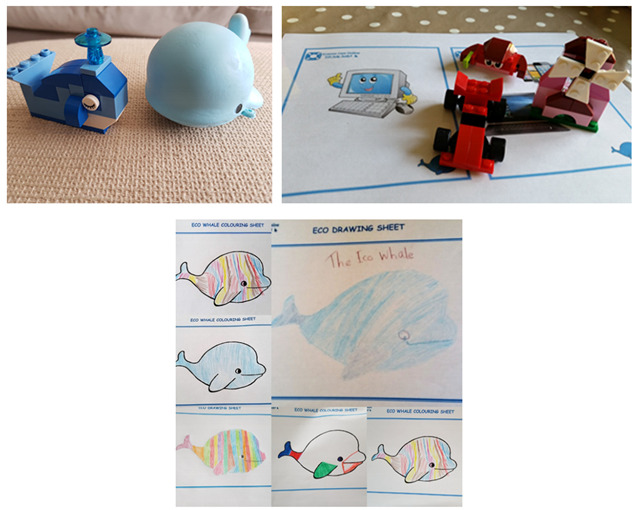

Box 4 **Child participant’s illustration of applying topical treatments within school.**

Picture shows process participant getting treatment from cupboard and applying her creams in the classroom (behind the desk).

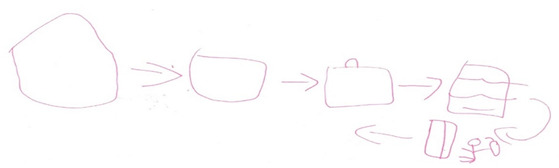

### 2.5. Data Analysis

With child participants’ and parents’ permission, interviews were digitally recorded and transcribed verbatim. Once transcribed and checked, audio recordings were deleted. Transcripts were assigned a study ID to ensure anonymisation of the data. We conducted an inductive thematic analysis [39] to analyse the interview data. One author (ET) read the transcripts multiple times in order to become familiar with the data and then coded the data line by line using NVivo version 12. Codes were derived inductively from the data and grouped together to produce an initial coding frame, which was reviewed and refined with other co-authors (KS, IM and MS). Then a detailed coding manual was created to allow systematic coding of the data. Codes were discussed regularly with all co-authors to iteratively develop and refine key themes arising from the data and to ensure a rich and diverse interpretation of the data (triangulation). A negative case analysis was carried out to ensure that all data were considered rather than selecting data that fitted with the authors’ viewpoints. Data collection stopped once we deemed data saturation for the main themes had been achieved (i.e., no new information emerged from the interviews).

### 2.6. Reflexivity

A reflexive stance was maintained throughout by taking into consideration the interviewers’ positioning as female research psychologists (ET and DG) and our involvement in the larger ECO intervention development project. The wider research team also consisted of a GP (MS), a Health Psychologist (IM), a research psychologist (KS), a dermatology nurse (SL) and a patient representative (AR), who are all female and some of whom are also mothers of children with eczema.

## 3. Results

### 3.1. Participant Characteristics

We conducted 14 semi-structured, face-to-face interviews between March and July 2018. Participants were predominantly girls (*n* = 9) and had mild to moderate eczema (*n* = 11). We interviewed children in all age groups from 6 years to 12 years; 8 participants were aged 6–9 years old and 6 were aged 10–12 years (Table 1). Parents were present during 9 interviews.

### 3.2. Key Themes

Inductive thematic analysis of interview data highlighted four main themes: (1) views about the nature of eczema; (2) positive effects of topical treatments; (3) perceived difficulties of applying topical treatments and what can make it easier; (4) desire for continued parental support with eczema management. Further analysis of the content of these overarching themes resulted in subthemes framed around the language of the child participants. We found that participants commonly called their topical treatments “creams”. They used this term to describe creams, lotions, gels or ointments and usually spoke about emollients rather than TCS. We explore each of the themes/subthemes in detail below and present selected quotes to illustrate each theme. Quotes are labelled with participant ID, age and parent-reported eczema severity.

#### 3.2.1. Views about the Nature of Eczema

“Eczema is like on/off, on/off”

We found that eczema was commonly viewed as short-term episodes that resolve but then flare up again rather than a long-term condition. Participants typically described their eczema as something that goes away but then starts again, is on and then off or moves to other parts of the body, not something that is constant.

*“Parent: I would say rather than it being a cycle that’s repeated, it’s more dependent on the weather.*

*C11: No. No, it’s a straight line.*

*Interviewer: What did you mean by ‘it’s a line’?*

*C11: That it doesn’t happen every single day, so it’s a new line. It’s a new time.”*

(C11, 6-year-old girl, mild eczema)

*“It keeps going up and down, so it has its times. It’s either the back of my knees because it’s quite sweaty and it’s quite itchy and everything, it doesn’t crack up like my hands and feet does, it’s just like little bits. So, it’s just quite flaky underneath, but then just sometimes underneath my knees flare up a lot, but then my hands get a lot better. So, it’s like on/off, on/off.”*

(C13, 12-year-old boy, moderate eczema)

“I want to find a solution to make my eczema go away”

Most children spoke about eczema as a temporary or passing phase that “you may grow out of”. Some older participants expressed disappointment that they had not grown out of it yet, either because of having heard of others who had grown out of it or because of a feeling that it must be possible to find a “solution”.

*“Some of my friends are saying that some of their relatives are having eczema, and just talking all about them, and saying that their eczema has gone away. Then that makes me feel kind of bad because some people’s eczema has gone away and mine hasn’t.”*

(C6, 8-year-old girl, severe eczema)

*“I would like to find a solution to put it all away, but that’s probably not going to happen, but it would be nice to have it gone. I’ve heard people saying they grow out of it, but I haven’t had that.”*

(C13, 12-year-old boy, moderate eczema)

There was some evidence of an emerging shift in mind-set away from seeking a cure to seeing eczema as manageable (i.e., it is possible to get control of eczema symptoms). Some older participants shared experiences of “looking after their eczema” effectively by using topical treatments and managing potential triggers.

*“It’s not too bad if you use the right cream and if you look after your eczema then it doesn’t really affect you much but if you don’t do anything about it then it can get worse.”*

(C9, 12-year-old girl, moderate eczema)

*“Most nights I was staying up all night, trying to slow down the sweating and everything. Now it’s been only one night every month or something, so it’s been a lot better. I think now it’s just because I’ve been taking care of myself a lot better. I’m bigger, I can look after myself a lot more now”*

(C13, 12-year-old boy, moderate eczema)

“Things that make my eczema worse”

Participants expressed further beliefs around identifying and managing triggers. Our data highlighted a predominant belief that eczema is made worse by external factors such as being outside, swimming and bathing and feeling hot and sweaty. Several participants described experiences where heat and sweat had made their eczema symptoms worse and their resultant focus on trying to find ways of keeping cool. Although it should be noted the predominance of this belief may have been influenced by the timing of the interviews in Spring and Summer. In contrast, some participants expressed that eczema “had a mind of its own” and expressed uncertainty about being able to identify and manage triggers.

*“In the daytime, if it’s really hot…sometimes, it starts to get a rash down my arms and it starts to itch a bit more, so I just go inside and get cold water, piece of paper towel on it. Then it starts to ease up a bit more”*

(C12, 11-year-old boy, moderate eczema)

*“It just becomes itchy whenever it wants. It has a mind of its own. It’s normally more like on the weekends when it’s really sunny and I’ve spent quite a lot of time on the trampoline.”*

(C3, 9-year-old girl, mild eczema)

#### 3.2.2. Positive Effects of Topical Treatments

“My creams soothe the itch and pain”

A dominant experience to emerge from the interviews was that using topical treatments can provide relief from eczema symptoms. Many participants described their eczema symptoms such as itch/pain as “annoying and frustrating” and disruptive to home and school life but generally felt that applying “creams” (predominantly emollients) was helpful in relieving those symptoms or “soothing the itch and pain”. Even participants, who expressed disliking their topical treatments in terms of texture or odour, felt that applying creams made them feel better. This suggests that perceiving topical treatments as soothing may be a potential facilitator of treatment adherence.

*“Kind of annoying because it gets in the way and kind of disturbs you when you’re trying to get to sleep, but it does get better when I just treat it and things like that, like once I put my—bought some cream and it feels better once I put that on.”*

(C5, 9-year-old girl, mild eczema)

*“Dry skin… it always itches all the time…I don’t like it…Itching it, but it’s really hard to stop itching it because in bed I scratch my feet sometimes, and then if I see scratches on them, and then when I put it [emollient] on it goes away…It helps—it helps your eczema and your dry skin.”*

(C4, 7-year-old boy, moderate eczema)

In addition to feeling that topical treatments help in reducing itch and pain, participants also commonly felt that emollients were effective in helping to reduce scratching. Participants reflected on how applying emollients created a physical “barrier” which prevented them from scratching because their skin felt slippery or they did not like the feel of the cream their hands.

*“C11: It basically stops me scratching when I have the cream on.*

*Parent: Is that because it reminds you that you shouldn’t scratch?*

*C11: No, I can’t scratch because it’s too slippery.”*

(C11, 6-year-old girl, mild eczema)

*“I feel happy and it’s helping me and then, when you go to bed or you’re awake, you don’t scratch that much because you have cream on it and when you have cream on it, you might not like the feel of the cream, so you stop itching it until the cream dries in.”*

(C14, 7-year-old girl, severe eczema)

#### 3.2.3. Perceived Difficulties of Applying Topical Treatments and What Can Make It Easier

“I find it difficult applying creams at school”

We found a general preference amongst participants towards “just wanting to apply creams at home”, which seemed to be due to feeling that their eczema was not bothersome during the school day or perceived difficulties around treatment application away from the home environment. Many participants described practical difficulties around the self-application of topical treatments at school. Practical difficulties included not being able to easily treat “hard to reach” patches under clothing and not wanting to ask the teacher to leave the classroom and disrupt schoolwork to apply their topical treatments, which were commonly stored in the school office or medical room. In some cases, the school did not seem to accommodate application of topical treatments.

*“Because I don’t use it anywhere else than my house I don’t mind. If I had to use it at school or something I wouldn’t want to use it… I don’t really like to use this one as much because it takes quite a long time to soak and because I normally wear trousers or shorts to school I can’t really get it there.”*

(C1, 8-year-old girl, severe eczema)

*“Sometimes bring cream down with me and we keep it in the [school] office and then I go and put it on when I need it… It’s okay but it kind of is—gets in the way of me missing out parts of my lessons.”*

(C5, 9-year-old girl, mild eczema)

In contrast, some participants shared experiences of easily accessible creams that were stored in easily reachable cupboards in or very near to the classroom (Box 4). It was noted that in these cases either a parent worked at the school or had set up a treatment plan in collaboration with the school, suggesting that an effective relationship between the school and parents may help to overcome such perceived difficulties and support treatment adherence away from home.

*“I get to school, I go into my classroom, then I sit down at my table and then I—and then we take the register. Then I start to get itchy so then I open the cupboard, sit down and put my cream on. Then I get back to my table. Because I sit right near the medical cupboard, I just get—I get my cream and I put it on wherever it’s itchy I normally just hide behind my table…I normally just go down here. Nobody can see me.”*

(C3, 9-year-old girl, mild eczema)

Participants also shared experiences of using strategies to reduce scratching at school, predominantly various distraction techniques, with mixed success. It appeared that some school environments may inadvertently have a disempowering effect on the child’s eczema management e.g., feeling stuck in hot, stuffy, carpeted classrooms.

*“I just start doing my work and then it makes me distracted and then I don’t feel like I’ve got to scratch.”*

(C14, 7-year-old girl, severe eczema)

*“I sometimes—like at school I put my cardigan on so I can’t [scratch]… It’s more itchy when it’s really hot, because like in my classroom it’s really hot. It’s more—it doesn’t happen when I’m cool, and my gym’s really cool.”*

(C3, 9-year-old girl, mild eczema)

*“It feels quite dry sometimes, and sometimes I’m really itchy at school, and the carpet sometimes makes my legs quite itchy. I tell my teacher, but she says that I should tell her later, so she doesn’t really help…To stop myself from itching, that’s very hard.”*

(C6, 8-year-old girl, severe eczema)

We also found perceived emotional difficulties around applying topical treatments at school such as feeling self-conscious and uncomfortable, especially during physical education classes and swimming lessons, as they felt this led to other children questioning what they were doing and making unwanted comments. It is interesting to note that even those participants who expressed generally positive experiences of applying treatment at school also reported emotional difficulties within certain contexts within the school day suggesting that treatment experiences differ within different environments which may affect treatment adherence at times when it is most needed.

*“If I’m on the other side of the room and I need to go up and get my cream, nobody looks at me then; it’s only after swimming when people look at me. After swimming at school they ask me; why do I have to put my cream on? I just find it really annoying… I don’t really like to put it on in front of people, but I kind of have to.”*

(C3, 9-year-old girl, mild eczema)

“Putting on creams is frustrating and slow”

Many participants viewed treatments as too time-consuming and spoke about frustration, competing priorities or forgetting to use their topical treatments. This was described within the context of having other things competing for their time.

*“Sometimes you just forget. It might be not having the time because we had lots of summer exams so we had to do lots of revision so I barely had any time.”*

(C9, 12-year-old girl, moderate eczema)

Some expressed frustration and annoyance more specifically about the slow absorption of treatments, in terms of having to “wait a long time for the creams to soak into the skin”.

*“I’d probably want to wait for the [emollient] kind of cream to soak in first and then put on sun cream but in the mornings I often don’t have time for that so I just want to put on one and go.”*

(C1, 8-year-old girl, severe eczema)

*“It’s quite greasy, so it’s hard to work with iPads, TV remotes and everything like that …the cream doesn’t go straight after you’ve rubbed it in. So if you touch it, it’s all greasy and everything….it takes a good 10, 15 min to dry off, so it’s quite annoying.”*

(C13, 12-year-old boy, moderate eczema)

“I don’t like how the cream feels on my skin”

As well as frustration with perceived slow absorption, participants also expressed mixed views about the texture, viscosity and odour of some topical treatments. A dominant view was that emollients feel slippery and sticky on the skin. Many participants described the emollients as thick and greasy and expressed a preference for “lighter” creams, although one participant expressed a preference for thicker cream as she felt they were more effective in helping her eczema. Some participants also described steroid creams as something that stung when applied and which have an unpleasant odour.

*“That one’s okay because it’s not so thick, but that one [emollient ointment]…I stick to things sometimes…If I’m just like in my pants and like someone’s doing my cream, it’s like I stick to the towel.”*

(C1, 8-year-old girl, severe eczema)

*“C8: I just didn’t like the feel of it on my arms; it was quite heavy and greasy.*

*Interviewer: What about the other cream that you liked?*

*C8: It was nice and light, isn’t it?”*

(C8, 12-year-old boy, moderate eczema)

*“I like that [steroid cream] helps my eczema, but sometimes it’s quite stingy when I put it on, and then sometimes I rub it off because it’s quite itchy.”*

(C6, 8-year-old girl, severe eczema)

“Making treatment times easier”

As well as the perceived difficulties of applying treatments or having them applied, participants also spoke about things they felt made treatment times easier, more fun and relaxing. A common approach was playful distraction techniques employed by parents that participants found helpful. For the younger children this included playing with a toy during treatment application and watching TV or having a story read to them whilst allowing creams to soak in. For older children there appeared to be a greater emphasis on role playing activities for example some participants spoke about treatment application as pampering or “spa” activities such as having massage or a spray tan (when using spray emollients). There was also a sense that as the child gets older, treatment becomes a routine and is perceived as an aspect of daily life or as “just something I just have to do”.

*“Dad gets the [emollient] and my [topical corticosteroid] and just gives me a massage into all the other places. I get special treatment every night!… It becomes one of those things at night, a massage parlour. He lies down, hands one after the other. Totally pampered.”*

(C12, 11-year-old boy, moderate eczema)

*“I do know more what to do now than when I was six or something, because now I’ve actually got into the routine of it so I know how to do it all.”*

(C13, 12-year-old boy, moderate eczema)

*“I just put it in my room so when I wake up I can see it and then it just sort of reminds me”*

(C4, 7-year-old boy, moderate eczema)

#### 3.2.4. Desire for Continued Parental Support with Eczema Management

“Sometimes I feel able to put my own cream on”

Many participants spoke about feeling able to apply creams themselves sometimes, but some younger participants said that they often forgot and needed reminders from their parents to apply their treatments. Others shared experiences of self-applying their topical treatment but commented on how they sometimes need help from parents with applying them in hard to reach places or when applying thicker emollients or steroid creams.

*“Usually when I’m itching my arms Mum tells me to go put it on. The rest of the time I usually forget.”*

(C4, 7-year-old boy, moderate eczema)

*“When it starts to flake up and it’s getting more dry skin underneath my nose, then mum just realises, and she says just to put some cream on.”*

(C12, 11-year-old boy, moderate eczema)

*“My mum helps me with my back because I can’t reach my back so… I remember when she was doing it when I was in Year 1, but from Year 2 onwards I did it on my own….I put this on my whole body before I go to school, because that sometimes stops me itching as much. Sometimes, not all the time because I am known as the scratcher because I always scratch! My mum does my back, but I do everything else.”*

(C3, 9-year-old girl, mild eczema)

“I can do it when I want to”

Recognising the benefits of taking responsibility for eczema management was evident amongst some older participants. Many felt that greater convenience was a particular benefit of self-application of treatment in terms of feeling able to apply their creams at a time that suited them rather than at a time that suited their parents.

*“I know what cream I can use, and I don’t have to wait for Mummy to be free.”*

(C6, 8-year-old girl, severe eczema)

*“It’s better because you haven’t got a certain time to do it. So when you’ve got some spare time, you can actually do it then, rather than stopping doing something you want to do…Mum says right, it’s got to be done by a certain time, so that gives me time to finish what I’m doing and then I can do it.”*

(C13, 12-year-old boy, moderate eczema)

## 4. Discussion

This qualitative interview study revealed novel insights into children’s views of eczema and implications for the perceived necessity of long-term (preventative) treatment, as well as perceived difficulties of children co-managing eczema at home and treatment adherence away from the home environment. Participants discussed beliefs in all the domains of the common-sense model [10], suggesting that, in addition to parents’ beliefs, children’s beliefs and perceptions may also influence treatment adherence and eczema co-management.

In terms of timelines, our findings suggest that children may view eczema as short-term (short episodes that resolve) and something you can outgrow or that can be cured rather than as a long-term condition. This may have implications for the perceived necessity of continuing treatment when eczema is seen as “resolved” i.e., regular emollient use to reduce flare ups rather than just symptom relief and may hinder the process of accepting a “control not cure” message and learning to self-manage eczema. This finding supports previous qualitative research highlighting children’s hopes for a miracle cure and how young people find the episodic nature of eczema challenging [25,26].

Our findings also suggest that children can find it difficult to manage their eczema at school, due to a lack of convenient access and appropriate spaces to apply topical treatments, and psychosocial consequences such as attracting unwanted attention from peers and feeling self-conscious. Previous research has highlighted a strong need for young people (over 13 years) with eczema to feel “normal” and not stand out amongst peers [40]. Our data support this previous finding and suggests this need is present at a younger age.

Increasing autonomy over eczema management was evident amongst study participants. Many were actively involved in applying treatments with their parents and some were self-applying treatments, managing triggers and findings ways to reduce scratching in certain contexts e.g., outside of the home environment. Our findings suggest that children welcome the benefits of greater convenience and feeling involved in their eczema management but can feel uncertain about eczema management. This suggests that the cognitive burden of treatment still resides with parents and that children in this age group may need support with taking steps towards transitioning to self-management.

As well as novel insights into the perceived barriers children face when applying treatments at school, we also highlighted other potential barriers to treatment adherence such as frustration with the time-consuming nature of treatment and disliking the “skin feel” of some topical treatments. In terms of potential facilitators, participants reflected on beneficial distraction techniques and attempts to establish treatment routines. Topical treatments (predominately emollients) were commonly seen as effective in terms of relieving symptoms of itch and pain. Associating emollients solely with symptom relief may present a potential barrier to accepting eczema as a long-term condition requiring ongoing regular use of emollients to keep control of eczema and prevent flare ups. Similar barriers and facilitators to treatment have been described in previous qualitative studies with parents of children with eczema [41,42,43,44,45,46,47,48]. Disliking treatments was highlighted in previous systematic review [49] which found that “just not liking it” contributed to treatment non-adherence, which suggests that non-adherence may not just be about illness/treatment beliefs, but often people just do not like taking treatments. In addition to discussing views and experiences around timelines, controllability and consequences, we also highlighted children’s beliefs about identity (annoying and frustrating symptoms of itch and pain) and causes (heat and sweat).

Schools and school nursing teams can play an important role in facilitating a realistic eczema plan in school for individual children to ensure they are able to manage their triggers and access their topical treatments and apply these with support and privacy. Health professionals could explain to children that eczema can be a long-term condition and episodic and that emollients can be used regularly to reduce flare-ups. It is also important to offer a choice of topical treatments e.g., if a child does not like texture or smell and ensure that parents understand they can seek alternatives if they child dislikes a particular cream. Supporting the transition from parental management to co-management to self-management could include developing own routine at a convenient time for the child, setting reminders, advice about applying topical treatments and choices available.

Our findings highlight children’s views and experiences of eczema and its treatment and provides valuable insight into potential treatment adherence barriers and facilitators and views about parent/child co-management, which is an un-researched area. Another strength of this study is that its trustworthiness has been ensured by adopting rigorous methods of data collection and analysis and reporting in line with Standards for Reporting Qualitative Research guidance [50]. A limitation is that although our study sample included a wide age range and heterogeneity in eczema severity, we did not collect further demographic data, such participant ethnicity, or recruit from more geographical locations, which may make these results less transferable.

## 5. Conclusions

Treatment adherence in children could be supported by reinforcing the fact that eczema is a long-term episodic condition, providing clear information about regular emollient use and practical advice to support co-management at home, and working with schools to facilitate effective topical treatment use when necessary.

## Figures and Tables

**Table 1:** Participants characteristics (n = 14).

	Number of Interviewees
**Child gender**	
Female	9
Male	5
**Child age**	
6 years	2
7 years	2
8 years	2
9 years	2
10 years	2
11 years	1
12 years	3
**Eczema severity ^1^**	
mild	5
moderate	6
severe	3

^1^ As defined by parent.

## Data Availability

Anonymized data are available on request from the corresponding author and with permission of the participants in the study. The data presented in this study are not publicly available due to ethical reasons (to protect the privacy of participants).
